# Pilose Antler Protein Relieves UVB-Induced HaCaT Cells and Skin Damage

**DOI:** 10.3390/molecules29174060

**Published:** 2024-08-27

**Authors:** Kaiyue Liu, Chenxu Zhao, Ke Zhang, Xiaoyue Yang, Ruyi Feng, Ying Zong, Zhongmei He, Yan Zhao, Rui Du

**Affiliations:** 1 College of Chinese Medicinal Materials, Jilin Agricultural University, Changchun 130118, China; 15870540735@163.com (K.L.); zs6607582@163.com (C.Z.); wojiaozhangkea@163.com (K.Z.); yxyaptx0713@163.com (X.Y.); fdd16639230916@163.com (R.F.); heather78@126.com (Z.H.); zhyjlu79@163.com (Y.Z.); 2 Jilin Provincial Engineering Research Center for Efficient Breeding and Product Development of Sika Deer, Jilin Agricultural University, Changchun 130118, China

**Keywords:** pilose antler, UVB, HaCaT cells, skin damage

## Abstract

Extended exposure to UVB (280–315 nm) radiation results in oxidative damage and inflammation of the skin. Previous research has demonstrated that pilose antler extracts have strong anti-inflammatory properties and possess antioxidant effects. This study aimed to elucidate the mechanism of pilose antler protein in repairing photodamage caused by UVB radiation in HaCaT cells and ICR mice. Pilose antler protein (PAP) was found to increase the expression of type I collagen and hyaluronic acid in HaCaT cells under UVB irradiation while also inhibiting reactive oxygen species (ROS) production and oxidative stress in vitro. In vivo, the topical application of pilose antler protein effectively attenuated UVB-induced skin damage in ICR mice by reducing interleukin-1β (IL-β), interleukin-6 (IL-6), and tumor necrosis factor-α (TNF-α) and inhibiting skin inflammation while alleviating UVB-induced oxidative stress. It was shown that pilose antler protein repaired UVB-induced photodamage through the MAPK and TGF-β/Smad pathways.

## 1. Introduction

The skin acts as the primary physical barrier for the body and is crucial in maintaining internal equilibrium [1]. External factors, such as physical and chemical agents, can cause damage to the skin. Skin aging is a widely researched topic, with ultraviolet radiation being the primary external factor contributing to it [2]. UVB (280–315 nm) is regarded as the most pivotal factor among these [3]. It can lead to skin photoaging and barrier damage, predominantly in the epidermis, and is accompanied by inflammatory responses that impair keratinocyte function [4]. Hyaluronic acid (HA) represents an important constituent of the extracellular matrix. Collagen is a significant biomarker of skin aging and is crucial for maintaining the skin’s structural integrity and elasticity [5]. Consequently, the measurement of dermal HA and collagen levels can serve as an effective indicator of the extent of photoaging in the skin. UVB radiation is absorbed by the skin and is a significant contributor to a spectrum of dermatological conditions. Furthermore, UVB exposure has been linked to the deterioration of the skin’s structural integrity through the induction of oxidative stress, DNA damage, and inflammation, which collectively give rise to a multitude of cutaneous complications. Prolonged exposure to UVB radiation results in the generation of reactive oxygen species (ROS), which can give rise to oxidative stress [6]. Additionally, UVB radiation causes direct cellular DNA damage. DNA damage can activate MAPK directly and indirectly through inflammatory factors such as interleukin-1 (IL-1), tumor necrosis factor-α (TNF-α), and interleukin- 6 (IL-6) [7]. This process results in the increased expression of intracellular matrix metalloproteinases (MMPs). Additionally, it inhibits the TGF-β/Smad signaling pathway and reduces type I pre-collagen synthesis. These effects ultimately lead to the dehydration, dryness, and desquamation of the skin, resulting in roughness, thickening, and loss of elasticity [8].

UVB-induced oxidative stress is regulated by the activities of catalase (CAT), superoxide dismutase (SOD), malondialdehyde (MDA), and glutathione peroxidase (GSH-Px) [9]. CAT is a crucial enzyme in the organism’s antioxidant defense system, while SOD is an important free radical scavenger. GSH-Px’s primary function is to scavenge lipid hydroperoxides, and MDA, a metabolite of oxygen radicals, is better suited to respond to the degree of lipid peroxidation in organisms [10]. However, excessive exposure of the skin to UV radiation results in the generation of ROS, which effectively destroys the skin’s antioxidant defense system and reduces the activity of SOD, CAT, and GSH-Px, leading to oxidative stress. Furthermore, ROS also react with fatty acids, resulting in lipid peroxidation [3]. ROS damage skin cells by oxidizing and promoting the synthesis of MMPs [11]. In addition to inducing oxidative stress, UVB radiation typically results in damage to the skin barrier. Skin barrier damage caused by UVB typically involves the disruption of hydration and keratinocyte function. Furthermore, UVB exposure has been demonstrated to induce oxidative damage through alterations in the expression of aquaporin protein-3 (AQP3), which ultimately results in the accumulation of reactive oxygen species and an increase in transepidermal water loss (TEWL) from the skin [12]. AQP3 is a transmembrane protein that primarily transports water molecules and glycerol, a key moisturizing factor for maintaining skin moisture [12]. Additionally, AQP3 promotes keratinocyte migration, proliferation, and differentiation in wound healing [13]. UV irradiation damages the skin through its relationship with ERK and AQP3 [12,14]. Additionally, filaggrin (FLG) and involucrin (IVL) are crucial factors for the skin barrier, while loricrin (LOR) is involved in the composition of the cornified envelope (CE). Studies have shown that UVB irradiation reduces the levels of FLG, IVL, and LOR proteins in HaCaT cells, leading to impaired skin barrier function [15].

Numerous natural products have demonstrated potent antioxidant and anti-inflammatory properties both in vitro and in vivo. Despite this, current research on anti-skin-photoaging primarily focuses on botanical drugs, with fewer studies on animal drugs. Deer antler protein, a protein-rich extract obtained from the antlers of sika deer and horse deer, exhibits a range of pharmacological activities, such as enhancing immunity [16], inhibiting inflammation [17], reducing oxidative stress [18], promoting collagen synthesis [19], and accelerating wound healing [20]. Research has shown that extracts of red ginseng and velvet antlers can prevent skin damage by enhancing the antioxidant defense system in UVB-irradiated HaCaT keratinocytes and SKH-1 hairless mice [21]. Additionally, a study demonstrated that UVB-induced skin photoaging is linked to oxidative stress, DNA damage, reduced skin elasticity, and inflammatory factors [22]. Given these findings, we propose that the repair of skin photoaging can be achieved through the inhibition of inflammatory factors, a reduction in oxidative stress, and the promotion of collagen synthesis. In this work, the study of pilose antler protein (PAP) repair of UVB-induced HaCaT cells and skin photoaging in mice was carried out by extracting sika deer antler proteins (SPs) and horse antler proteins (HPs).

## 2. Results

### 2.1. PAP Treatment Restored UVB-Induced HaCaT Cell Viability and Reduced HaCaT Cell Death

**Figure 1:**
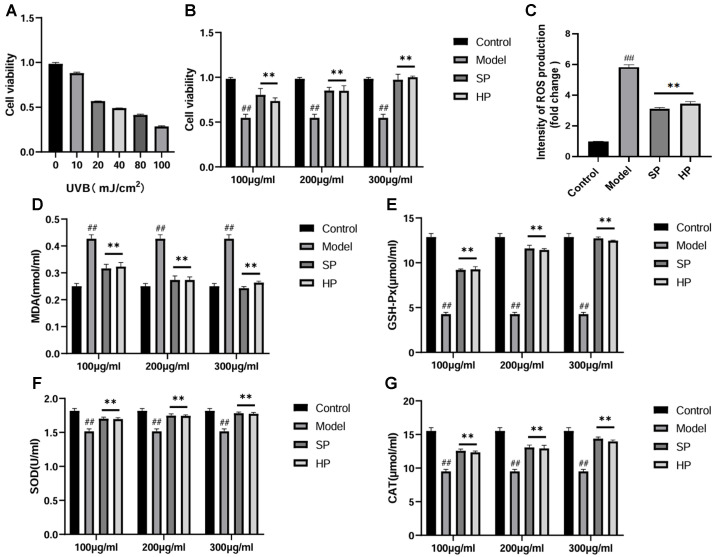
PAP increases UVB-induced HaCaT cell viability and decreases cellular oxidative stress levels (control: without treatment; model: cells + UVB; SP: cells + UVB + sika deer antler proteins; HP: cells + UVB + horse antler proteins). (**A**) The cell viability of HaCaT cells exposed to UVB with different intensities (*n* = 6); (**B**) the cell viability of HaCaT cells exposed to UVB and treated with SPs and HPs. (*n* = 6); (**C**) quantitative analysis of reactive oxygen species (ROS) production (*n* = 3); (**D**–**G**) the effects of SPs and HPs on superoxide dismutase (SOD), catalase (CAT), and glutathione peroxidase (GSH-Px) activities and malondialdehyde (MDA) content (*n* = 3). ** *p* < 0.01 versus the model group, ## *p* < 0.01 versus the control group.

As the UVB irradiation dose increased, the cell survival rate decreased in a radiation-dose-dependent manner (Figure 1A). For subsequent experiments, we selected an LD50 of 40 mJ/cm^2^. All administered treatments showed significant repair effects on cell photodamage at concentrations of 100–300 μg/mL (*p* < 0.05) in comparison to the model group (Figure 1B). The repair effects exhibited a dose-dependent increase. The results showed that SPs and HPs were highly protective for the survival of UVB-induced HaCaT cells, in which a concentration of 300 μg/mL resulted in a cell survival rate that was only slightly lower than that of the normal group. This study found that antler proteins were effective in repairing the damage caused by UVB to HaCaT cells.

### 2.2. PAP Reduced Oxidative Stress in HaCaT Cells

UVB irradiation significantly induced excessive ROS production (Figure 1C) and increased MDA levels (Figure 1D) in the cells. Furthermore, a notable decline in GSH-Px, SOD, and CAT activities was observed (Figure 1E–G). Nevertheless, the PAP intervention led to a notable reduction in ROS and MDA levels (Figure 1C,D) while restoring the cellular levels of GSH-Px, SOD, and CAT (Figure 1E–G). The findings indicate that PAP mitigates the impact of UVB-induced reactive oxygen species in HaCaT cells and reduces cellular oxidative stress.

### 2.3. PAP Increased Type I Collagen (ColⅠ) and Hyaluronic acid (HA) in UVB-Irradiated HaCaT Cells

**Figure 2:**
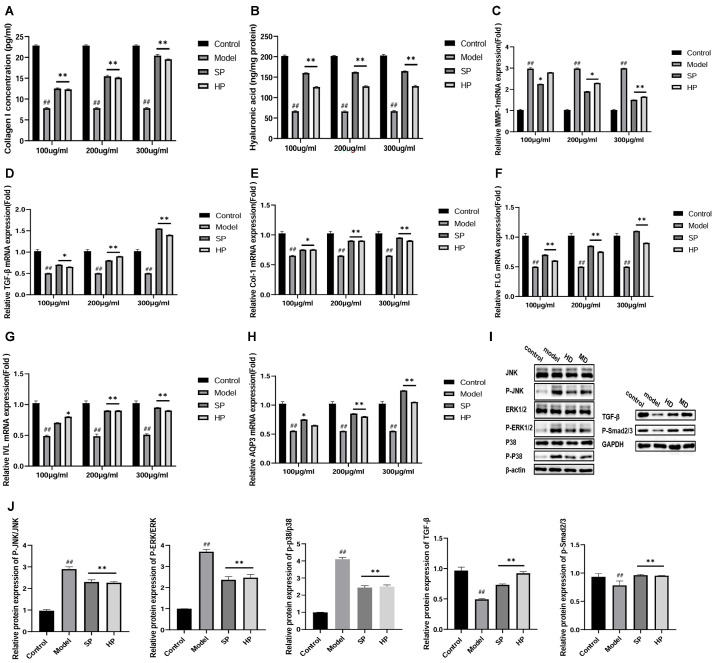
PAP improves collagen synthesis and barrier function in UVB-induced HaCaT cells. (**A**,**B**) The effects of SPs and HPs on type I collagen (ColⅠ) and hyaluronic acid (HA) content (*n* = 3); (**C**–**H**) changes in the expression levels of genes associated with the TGF-β/Smad pathway and skin repair. Real-time fluorescence quantitative PCR showing the mRNA expression of matrix metalloproteinase 1 (MMP1), transformation growth factor-β1 (TGF-β1), type I collagen (ColⅠ), filaggrin (FLG), involucrin (IVL), and aquaporin protein-3 (AQP3) (*n* = 6); (**I**,**J**) representative images of protein blots of MAPK and TGF-β/Smad pathway-related proteins and quantitative analysis of the images (*n* = 3). * *p* < 0.05, ** *p* < 0.01 versus the model group, ## *p* < 0.01 versus the control group.

Collagen I is the most abundant protein in the human body. A significant reduction in collagen I content was demonstrated in the model group in reference to the control group (Figure 2A). However, the SP and HP treatments effectively alleviated the decrease in collagen I content (*p* < 0.01). In addition, HA helps to maintain skin moisture, promote cell proliferation, and induce cell differentiation. The HA content was markedly diminished in the model group in comparison to the control group (Figure 2B). However, the administered treatments effectively increased the HA content (*p* < 0.01), with the most significant effect observed in the SP group. These findings indicate that deer PAP can enhance cellular repair in HaCaT cells following their exposure to UVB radiation by increasing type I collagen synthesis and moisturization.

### 2.4. PAP Regulates the Expression of MMP-1, TGF-β1, Col Ⅰ, FLG, IVL, and AQP3 to Repair Photoaging in HaCaT Cells

The mRNA expression level of MMP-1 in HaCaT cells increased 2-fold after UVB exposure compared to the normal group (Figure 2C). Only SPs demonstrated a notable impact on the mRNA expression level of MMP-1 at all doses (*p* < 0.05). UVB irradiation significantly decreased the mRNA expression of collagen I and TGF-β1 (Figure 2D,E). In contrast, both drug administration groups increased the mRNA expression of collagen I and TGF-β1 (*p* < 0.05 or *p* < 0.01). The group that received a high dose demonstrated a more pronounced alteration in the expression of TGF-β1 relative to the control group. Both SPs and HPs demonstrated notable impacts on the mRNA expression levels of FLG, IVL, and AQP3 (Figure 2F–H). The results suggest that PAP can improve hydration and promote collagen synthesis.

### 2.5. Effects of PAP on MAPK and TGF-β/Smad Pathways in UVB-Induced HaCaT Cells

The ERK, JNK, and p38 proteins are crucial signaling molecules for cell proliferation, differentiation, and apoptosis in the MAPK pathway. In skin tissues, UVB radiation significantly increased the phosphorylated expression of these proteins. However, PAP treatment effectively inhibited the phosphorylation of MAPKs (Figure 2I,J). Smad is a cytoplasmic transcription factor that is activated by TGF-β and leads to the production of type I collagen in dermal fibroblasts. The dosed group showed an increase in TGF-β1 protein expression, while UVB irradiation resulted in a decrease (*p* < 0.01). Additionally, p-Smad 2/3 protein expression was significantly reduced by UVB irradiation (*p* < 0.01) (Figure 2I,J). However, the administration of the drug inhibited the reduction in protein expression. It seems that UVB promotes not only the increased expression of MAPK kinases but also the increased activation (phosphorylation) of kinases. Nevertheless, SPs and HPs attempt to counteract these effects, thereby alleviating oxidative stress and inflammation in the skin.

### 2.6. PAP Recovered UVB-Induced Skin Damage and Collagen Degradation

**Figure 3:**
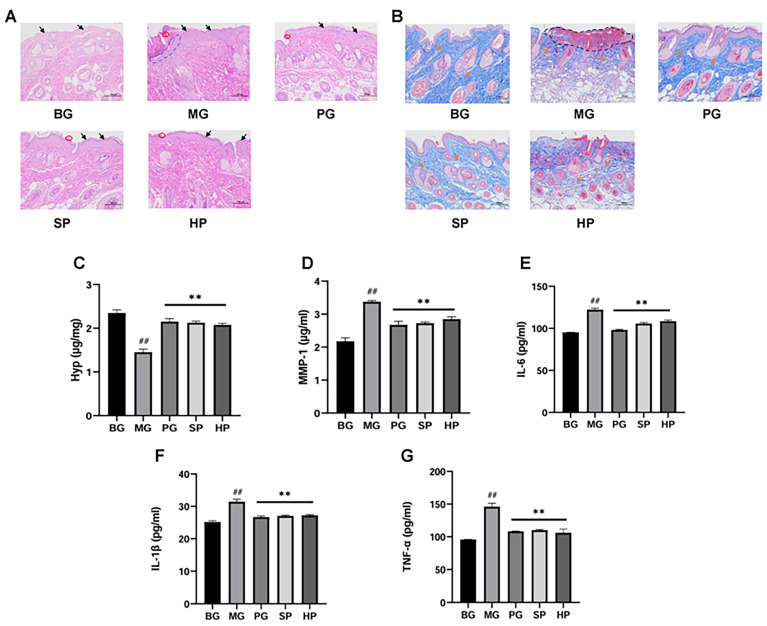
PAP improves epidermal thickening, collagen deposition, and inflammation of the skin caused by UVB. The groups were the normal group (BG), UVB model group (MG), 10 mg/mL sika deer antler protein treatment group (SP), 10 mg/mL horse antler protein treatment group (HP), and VC positive treatment group (PG). (**A**) HE staining of skin tissue (*n* = 3, scale bar = 100 µm). Black arrows indicate epidermal thickness. The blue dashed line indicates unhealed gaps. Red circles indicate diffuse inflammation. (**B**) Masson staining of skin tissue (*n* = 3, scale bar = 100 µm). Orange arrows indicate collagen fiber alignment. The black dotted line indicates decreased collagen fibers. (**C**) The change in Hydroxyproline (Hyp) levels in skin tissues (*n* = 3); (**D**) the change in MMP-1 levels in skin tissues (*n* = 3); (**E**–**G**) expression levels of inflammatory factors IL-6, IL-1β, and TNF-α in serum (*n* = 3). ** *p* < 0.01 versus the model group, ## *p* < 0.01 versus the control group.

Compared to the normal group (BG: without treatment), the UVB model group (MG: UVB radiation) exhibited irregular epidermal thickening (black arrows), a damaged stratum corneum (blue dotted line), abnormal collagen fiber arrangement (orange arrows), decreased collagen content (orange arrows and black dotted line), and inflammatory cell infiltration (red circles) (Figure 3A,B). The results indicate that the mouse skin photoaging model was successfully established, and the application of PG, SPs, and HPs significantly improved the pathological features of photoaging on the back skin of mice. The epidermal thickness in each group of mice was significantly reduced, and the structure of the epidermis and dermis became clearer, with a slight infiltration of inflammatory cells. The collagen fibers were more tightly arranged, and the collagen content was significantly increased in each dosing group, resulting in a significant improvement in skin fibrosis (Figure 3A,B). Therefore, PAP can alleviate the epidermal thickening and collagen degradation induced by UVB irradiation.

### 2.7. PAP Increased Hydroxyproline (Hyp) Content, Decreased MMP-1 Content in Skin Tissue, and Inhibited the IL-6, IL-1β, and TNF-α Concentrations in Serum

Hyp is a key component of collagen and serves as an important indicator of collagen metabolism and fibrosis. A significant deficit of Hyp content was observed in the MG group in comparison to the BG group (Figure 3C). Conversely, a notable elevation in the expression of MMP-1 was evident in the MG group (Figure 3D). However, PG, SP, and HP could enhance the levels of Hyp (Figure 3C) and reduce the expression of MMP-1 (Figure 3D). IL-6, TNF-α, and IL-1β play pivotal roles in initiating inflammatory responses. The concentrations of IL-6, IL-1β, and TNF-α were considerably elevated in the MG group (Figure 3E–G), while they were significantly lower in each of the administered groups. No notable discrepancy was observed between the administered group and the positive group in their effects on IL-1β and TNF-α. The results indicate that PAP can reduce cellular photoaging damage by restoring the decreased collagen content in mouse skin caused by UVB radiation. This is consistent with the results of Masson staining. Additionally, PAP can repair skin damage by inhibiting the expression of inflammatory cytokines.

### 2.8. PAP Reduced Oxidative Stress in UVB-Irradiated Mice

**Figure 4:**
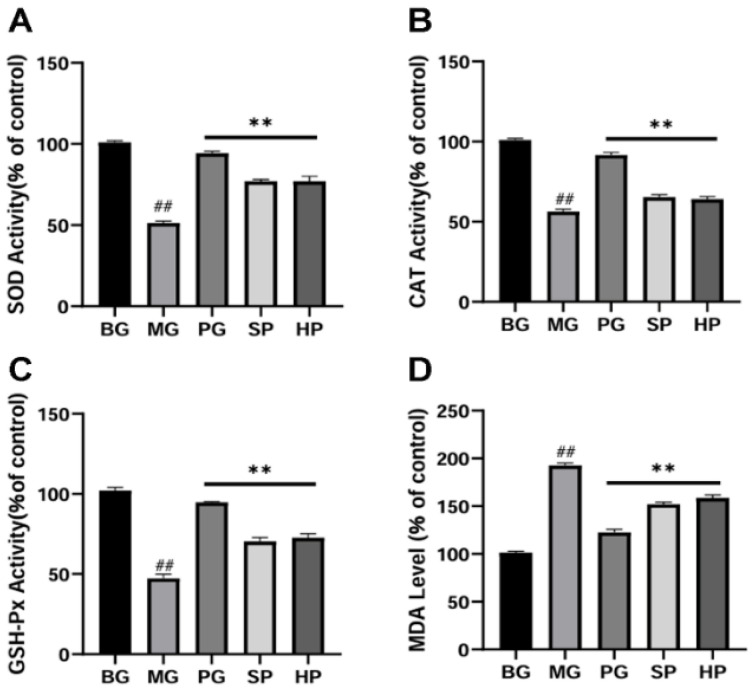
SPs and HPs alleviate UVB-induced oxidative stress in ICR mouse skin. (**A**) The SOD activity in skin tissues (*n* = 3); (**B**) the CAT activity in skin tissues; (**C**) the GSH-Px activity in skin tissues (*n* = 3); (**D**) the MDA level in skin tissues (*n* = 3). ** *p* < 0.01 versus the model group, ## *p* < 0.01 versus the control group.

UVB radiation led to a reduction in the activity of SOD, CAT, and GSH-Px (Figure 4A–C) and caused an increase in MDA content (Figure 4D) in mouse skin. Nevertheless, SPs and HPs markedly diminished oxidative stress in the skin, a finding that aligns with the in vitro outcomes. These findings suggest that PAP can alleviate oxidative stress and promote the repair of skin photoaging damage.

### 2.9. PAP Regulates the Expression of MMP-1, TIMP-1, Col-Ⅰ, Col-Ⅲ, and TGF-β1 to Repair Photoaging in Mice

**Figure 5:**
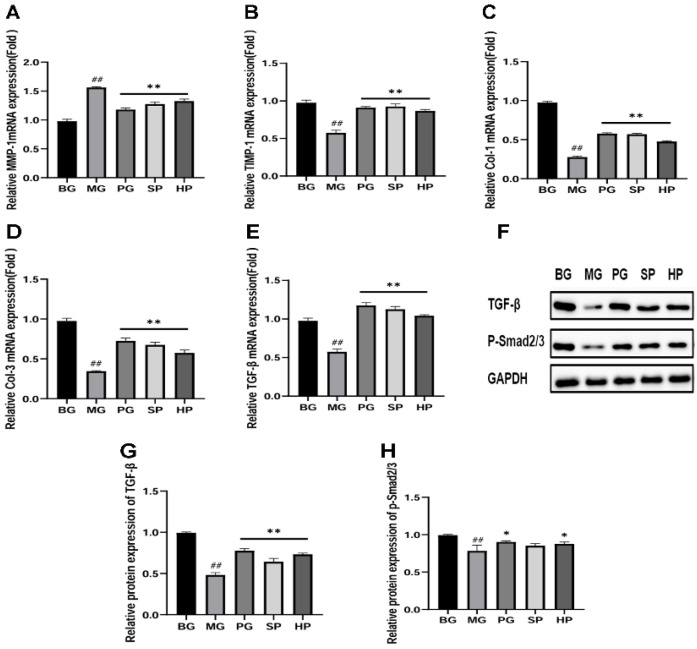
The expression of related genes and proteins in mouse skin tissues (*n*  =  3). (**A**) The mRNA level of MMP-1; (**B**) the mRNA level of tissue inhibitor of metalloproteinase 1 (TIMP-1); (**C**) the mRNA level of ColⅠ (*n* = 3); (**D**) the mRNA level of collagen type III (Col III); (**E**) the mRNA level of TGF-β1; (**F**) protein banding; (**G**) quantitative analysis of TGF-β1 protein; (**H**) quantitative analysis of p-Smad 2/3 protein. * *p* < 0.05, ** *p* < 0.01 versus the model group, ## *p* < 0.01 versus the control group.

The RT-qPCR results indicated that UVB radiation caused an increase in the mRNA level of MMP-1 in the skin (Figure 5A). This situation was alleviated in each dose administration group (*p* < 0.01). Additionally, UVB irradiation reduced the mRNA expression of tissue inhibitor of metalloproteinase 1 (TIMP-1), collagen I, collagen type III (collagen III), and TGF-β1 (Figure 5B–E). The administration groups were able to significantly increase the mRNA expression of TIMP-1, TGF-β1, and collagen I and III (*p* < 0.01). Nevertheless, no discernible discrepancy was observed between the SP and PG groups.

### 2.10. PAP Upregulated TGF-β and p-Smad 2/3 Expression in Skin Tissues

This study investigated whether deer antler proteins could activate the TGF-β pathway and accelerate the synthesis of type I pre-collagen via the intracellular transcription factor Smad. The findings demonstrated that UVB radiation reduced the expression of TGF-β and p-Smad 2/3 proteins, while the administered group restored the reduced protein expression (Figure 5F–H).

## 3. Discussion

Repeated or long-term exposure to UV radiation can damage the skin barrier, causing dryness, dehydration, redness, darkening, roughness, thickening, and loss of elasticity [23]. UVB primarily affects the epidermis, and HaCaT cells are the most important cells in the epidermis [24]. Irradiation of HaCaT cells with UVB is a commonly used method of constructing a cellular model of photoaging [25]. The TGF-β/Smad and MAPK signaling pathways influence collagen metabolism and are involved in the process of photoaging [26]. However, to more accurately mimic normal human skin, ICR mice were chosen for this experiment. Natural compounds have been found to have photoprotective effects against UVB radiation-induced skin damage due to their bioactive substances [27,28,29]. The mechanism of action of deer antlers on epidermal cells and mouse skin is not clear, despite its known anti-photoaging effects.

UVB radiation induces oxidative stress and inflammatory responses in the skin [30,31]. Previous studies have reported that pro-inflammatory cytokines, such as TNF-α and IL-6, exert a pivotal influence in the early phase of the skin photoaging response [32]. UVB-induced oxidative stress activates the MAPK pathway, leading to increased phosphorylation levels of JNK, ERK, and p38 proteins [33]. UVB-induced ROS are a pivotal factor in stimulating melanogenesis through the MAPK signaling pathway [34]. Additionally, UVB irradiation reduces collagen production by inducing MMPs, leading to the degradation of collagen I and III, ultimately resulting in wrinkles [35]. It is worth noting that Hyp is the signature component of collagen [36]. Hyaluronic acid levels are indicative of skin hydration [37], while AQP3 is a crucial moisturizing factor that helps maintain skin moisture [38]. Additionally, TGF-β1 is a multifunctional cytokine with a substantial impact on collagen and elastin synthesis [39].

In conclusion, antler proteins can effectively restore HaCaT cell activity, reduce ROS levels, increase SOD, GSH-Px, and CAT enzyme activities, and reduce MDA content, thus alleviating cellular oxidative stress. Additionally, deer antler proteins can increase Hyp and HA contents in cells, increase the expression levels of FLG, IVL, AQP3, type I collagen, and TGF-β1 mRNA, and decrease the expression levels of MMP-1 mRNA. This study also investigated the effects of deer antler proteins on photoaging in ICR mice. The results demonstrated that deer antler proteins effectively reduced epidermal thickening and collagen loss in photoaging mice. Additionally, the study found that deer antler proteins increased the Hyp content, which was consistent with the upregulation of mRNA levels of collagen I and III. TGF-β1, MMP-1, and TIMP-1 are closely related to collagen synthesis. Deer antler proteins can reduce the content and mRNA expression level of MMP-1, increase the mRNA expression of TGF-β1 and TIMP-1, and alleviate inflammation and oxidative stress in vivo. UVB-induced inflammation and oxidative stress can activate the MAPK pathway, leading to an increase in the phosphorylation levels of p38, ERK, and JNK proteins. However, antler proteins have been shown to effectively inhibit this protein phosphorylation. The biological effects on living organisms and the primary mechanism require further investigation.

## 4. Materials and Methods

### 4.1. Materials and Reagents

Sika deer antlers and horse deer antlers (Cervus nippon Temminck and Cervus elaphus Linnaeus) were purchased from Shuangyang Deer Farm (Jilin, China). HaCaT cell lines were purchased from Wuhan Saios Biotechnology Co., Ltd. (Wuhan, China).

Dulbecco’s modified eagle medium (DMEM medium), fetal bovine serum (FBS), and trypsin were provided by Thermo Fisher Scientific Co., Ltd. (Waltham, MA, USA). Vitamin C (VC) and Sodium chloride injections were provided by Solarbio (Beijing, China). Cell Counting Kit-8 (CCK-8) was obtained from Sigma (St. Louis, MO, USA). Reactive oxygen (ROS) was obtained from Beyotime (S0033S, Shanghai, China). Superoxide dismutase (SOD, A001-3-2), catalase (CAT, A007-1-1), glutathione peroxidase (GSH-Px, A005-1-2), malondialdehyde (MDA, A003-1-2), and Hyp (A030-2-1) were acquired from Nanjing Jiancheng Institute of Biological Engineering. (Nanjing, China). Type I collagen (MM-2023H1) and hyaluronic acid (MM-0358H1) Elisa Kits (human) and IL-6 (MM-0163M1), IL-1β (MM-0040M1), TNF-α (MM-0132M1) and MMP-1 (MM-61512R1) (mouse) Elisa Kits were provided by Jiangsu Meimian Industrial Co., Ltd. (Yancheng, China). All chemical reagents used were analytical grade.

### 4.2. Preparation of Pilose Antler Protein

Pilose antler protein (PAP) was prepared following the method in [19] with minor modifications. The protein was extracted using the ultra-micro-milling method with a material–liquid ratio of 1:10 and glacial acetic acid (with double-distilled water added to maintain pH = 4). The mixture was left overnight at 4 °C and then subjected to centrifugation at 4 °C and 5000 rpm/min for 10 min the following day. The resulting supernatant was collected. The dialysis method used was consistent with the study. The supernatant was desalted by dialysis in a 1 kD dialysis bag, filtered, concentrated in a rotary evaporator, freeze-dried, and stored at 4 °C. Sika deer antler proteins (SPs) and horse antler proteins (HPs) were obtained.

### 4.3. Cell Culture

HaCaT cells were maintained in DMEM medium containing 10% FBS and maintained in a CO_2_ incubator at 37 °C (St. Louis, MO, USA). The cell viability assay was conducted in accordance with the instructions provided by the CCK-8 kit manufacturer.

### 4.4. UVB Irradiation and Drug Therapy

To determine the optimal radiation dose for the UVB irradiation model, HaCaT cells were inoculated into 96-well microtiter plates at a concentration of 1 × 10^4^ cells/mL. Following incubation for a period of 24 h, the cells were rinsed twice in PBS (pH = 7.4) and irradiated with a UVB lamp (230–320 nm, 620 μW/cm^2^) at varying doses (0, 10, 20, 40, 80, 100 mJ/cm^2^). The optimal radiation dose was selected based on the assessment of cell viability.

To test the reparative efficacy of PAP on the survival of UVB-exposed cells, we exposed HaCaT cells to optimal doses of radiation. We then added DMEM medium enriched with 10% FBS and varying concentrations of PAP (100, 200, 300μg/mL of SPs and HPs) and cultured them for 24 h. The control group was maintained under identical conditions, excluding UVB exposure and PAP, while the UVB model group received the same dose of UVB exposure without PAP. The experimental groups received the same dose of UVB exposure with varying concentrations of PAP. Finally, cell viability was determined. The specific groups are shown in Table 1 below.

### 4.5. ROS Assay in HaCaT Cells

ROS content was detected by the chemiluminescence method using the fluorescent probe 2′,7′-Dichlorodihydrofluorescein diacetate (DCFH-DA). Cells were inoculated into 12-well culture plates at a density of 5000 cells/cm^2^, followed by cell culture and grouping as in Section 4.4. After UVB irradiation, a variety of concentrations of PAP were used in a co-culture of HaCaT cells for 24 h. Photographs were then taken using an inverted fluorescence microscope, with subsequent analysis of the fluorescence intensity conducted via the Image J software 8.0.

### 4.6. Analysis of SOD, GSH-Px, CAT, and MDA Contents in HaCaT Cells

SOD viability was determined using the water-soluble tetrazolium salt-1 (WST-1) method. CAT activity was determined by the ammonium molybdate method. GSH-Px activity was determined by a Colorimetric method. MDA activity was determined by the thiobarbituric acid (TBA method). To obtain the supernatant, the cells were lysed by sonication and then centrifuged at 12,000 rpm for 10 min. The total protein concentration present in the cell supernatants was measured using a BCA protein assay kit (Beyotime, Shanghai, China). The obtained supernatants were used for the assay of MDA, SOD, GSH-Px, and CAT. All metrics were measured in accordance with the specifications outlined by the manufacturer (Nanjing Jiancheng Institute of Bioengineering, Nanjing, China).

### 4.7. Detection of Type I Collagen and Hyaluronic Acid in HaCaT Cells

Cells were prepared and grouped as described in Section 4.4. They were then cultured for 24 h after drug administration, and the supernatant was isolated through centrifugation at 3000 rpm for 10 min. The collagen type I and hyaluronic acid contents in the cell supernatant were quantified through an ELISA kit (Meimian, Yancheng, China) following the provided instructions.

### 4.8. Real-Time qPCR (RT-qPCR) Analysis

The total RNA was extracted using a commercially available extraction kit (Takara, Kyoto, Japan), followed by cDNA synthesis using the super mix reverse transcription method. Real-time quantitative polymerase chain reaction was conducted via the SYBR Green qPCR master mix. The PCR primers were designed and synthesized by Shenggong Bioengineering (Changchun, China). Please refer to Table 2 for the primer sequences and the amount of each reagent used.

### 4.9. Western Blotting

A total protein extraction kit (BestBio, Beijing, China) was employed to extract the cell proteins, and a BCA kit (Beyotime, China) was utilized to quantitate them. The separation of the proteins was conducted through the utilization of sodium dodecyl sulfate–polyacrylamide gel electrophoresis (SDS-PAGE) gels. A total of 25 µg of protein was isolated per sample and resolved on SDS-PAGE gels. Subsequently, the transfer to polyvinylidene fluoride (PVDF) membranes was facilitated. After incubating the membrane with 5% skimmed milk for 2 h at room temperature, the primary antibody was added and incubated overnight at 4 °C, followed by the corresponding secondary antibody at room temperature the next day. Protein bands were analyzed with enhanced chemiluminescence reagents, and images were acquired using a ChemiDoc MP Imaging System (Bio-Rad Laboratories, Hercules, CA, USA). The specific antibodies utilized in the experiment are presented in Table 3 below.

### 4.10. Animals and Treatments

Fifty ICR mice of both sexes were purchased from Changchun Yisi Experimental Animal Technology Co., Ltd. (Changchun, China). All ICR mice were maintained under standard conditions, with a temperature of 25 ± 2 °C and a relative humidity of 50 ± 5%. The light/dark cycle was set at 12 h/12 h. The animals were acclimated to the environment before the experiment. All animal experiments were conducted according to the guidelines of the Declaration of Helsinki and the Animal Research Committee guidelines of Jilin Agricultural University (Jilin, China, Certificate number: SYXK(Ji)2018-0023; Ethics number: 20211011003).

**Figure 6:**
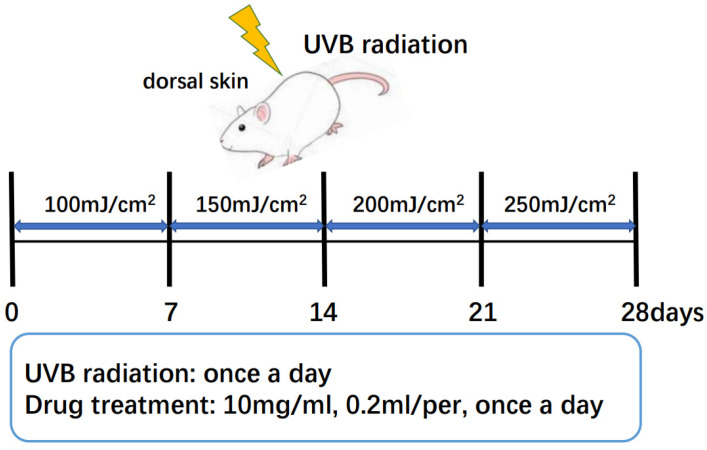
UVB radiation and treatment in mice.

All animals were randomly divided into 5 groups (10 ICR mice per group), with males and females in separate cages. The groups were the normal group (BG), UVB model group (MG), 10 mg/mL sika deer antler protein treatment group (SP), 10 mg/mL horse antler protein treatment group (HP), and VC positive treatment group (PG). The mice were exposed to UVB radiation for four weeks, except for the control group. During irradiation, the mice were immobilized. The dose of UVB radiation was 100 mJ/cm^2^ in the first week, 150 mJ/cm^2^ in the second week, 200 mJ/cm^2^ in the third week, and 250 mJ/cm^2^ in the fourth week. After irradiation, the drug was applied once a day at a concentration of 10 mg/mL and 0.2 mL per mouse (Figure 6). The BG and MG groups had the same dose of saline applied. After the last day of irradiation, the mice were humanely euthanized, and their dorsal skin tissues were acquired and stored at −80 °C.

### 4.11. Histological Analysis

Dorsal skin tissues were collected, fixed in 10% formalin, paraffin-embedded, and cut into 5 μm sections. The skin samples were processed for histological examination using hematoxylin and eosin (H&E) and Masson’s trichrome solutions. The epidermal thickness and collagen content of the skin were quantified using Image J software.

### 4.12. Measurement of IL-6, TNF-α, and IL-1β in Serum

ELISA kits were supplied by Jiangsu Meimian Industrial Co., Ltd. (Yancheng, China). Cytokine levels (IL-6, TNF-α, and IL-1β) were measured in accordance with the prescribed methodology.

### 4.13. Measurement of Hyp and MMP-1

Hyp was measured by relevant assay kits from Nanjing Jiancheng Institute of Biological Engineering (Nanjing, China). The Hyp content was determined by the alkaline hydrolysis method. The matrix metalloproteinase-1 (MMP-1) level in skin tissues was measured by relevant assay kits (Jiangsu Meimian, Yancheng, China).

### 4.14. Analysis of SOD, GSH-Px, CAT, and MDA Contents in Mice

Skin tissue samples were processed and assayed for SOD, MDA, CAT, and GSH-px in accordance with the manufacturer’s instructions (Nanjing Jiancheng Bioengineering Institute, Nanjing, China). These indicators were tested in the same way as in Section 4.6.

### 4.15. Real-Time qPCR (RT-qPCR) Analysis of mRNA in Skin Tissues

The subsequent experimental steps are the same as in Section 2.8. The primer sequences and the amount of each reagent used are shown in Table 4.

### 4.16. Western Blotting

The BestBio Total Protein Extraction Kit (Shanghai, China) was used to extract skin tissue proteins, and the protein concentration was quantified with the use of the BCA kit (Beyotime, China). Subsequently, the blots were cultured with the specific primary antibodies for evaluation. The blots were cleaned and re-incubated with the appropriate secondary antibodies conjugated with horseradish peroxidase, and chemiluminescence detection was performed. The experimental steps are the same as in Section 4.9. The antibodies used in the experiment are shown in Table 3.

### 4.17. Statistical Analysis

The image data underwent quantitative analysis using the ImageJ 8.0 software. All statistical data are presented as mean ± standard deviation (SD) and were visualized using GraphPad prism software (Version 8.0, La Jolla, CA, USA). A one-way analysis of variance (ANOVA) was employed to ascertain the dissimilarities between the groups. The significance levels of results considered statistically significant are indicated as follows: * *p* < 0.05, ** *p* < 0.01, # *p* < 0.05, ## *p* < 0.01.

## 5. Conclusions

In conclusion, this study demonstrated that PAP could protect HaCaT cells from injury after UVB radiation and alleviate oxidative stress effectively by reducing ROS levels, enhancing antioxidant enzyme activities, and decreasing MDA levels. Additionally, PAP increases the expression of collagen I, HA, and Hyp and suppresses the secretion of IL-1β, TNF-α, and IL-6, thereby inhibiting the UVB-induced inflammatory response and increasing the collagen content. This study found that antler proteins from sika deer and horse deer can repair skin photoaging through the MAPK and TGF-β/Smad signaling pathways.

## Figures and Tables

**Table 1:** Experimental grouping of HaCaT cells.

Group	UVB Radiation	Drug Concentration
Control	0	0
Model	40 mJ/cm^2^	0
SP	40 mJ/cm^2^	100 μg/mL
SP	40 mJ/cm^2^	200 μg/mL
SP	40 mJ/cm^2^	300 μg/mL
HP	40 mJ/cm^2^	100 μg/mL
HP	40 mJ/cm^2^	200 μg/mL
HP	40 mJ/cm^2^	300 μg/mL

**Table 2:** Primer sequences for HaCaT cells.

Primer Name	Sequence	Length
IVL-F	TTCCTCCTCCAGTCAATACCCATC	24
IVL-R	GCAGTCCCTTTACAGCAGTCATG	23
FLG-F	CTCATCACAGCCACACCACATC	22
FLG-R	GCCATCTCCTGATTGTTCCTTGTC	24
ColI-F	GGCAAAGAAGGCGGCAAAGG	20
ColI-R	GGAGCACCAGCAGGACCATC	20
TGF-β1-F	GCAACAATTCCTGGCGATACCTC	23
TGFβ1-R	CCTCCACGGCTCAACCACTG	20
MMP1-F	TTACACGCCAGATTTGCCAAGAG	23
MMP1-R	TCAGAGGTGTGACATTACTCCAGAG	25
AQP3-F	TGTGCTTCCTGGCTCGTGAG	20
AQP3-R	GCTGGTTGTCGGCGAAGTG	19
GAPDH-F	ACCCACTCCTCCACCTTTGAC	21
GAPDH-R	TCTACCACCCTGTTGCTGTAG	21

**Table 3:** Antibodies for Western blotting assay.

Name	Company
Rabbit Anti-JNK, ERK, P38 Monoclonal Antibody	Cell Signaling Technology (Danvers, MA, USA)
Rabbit anti-phosphorylated JNK, ERK, P38 polyclonal antibody	Cell Signaling Technology
Rabbit anti-β-actin polyclonal antibody	Cell Signaling Technology
Rabbit anti-GAPDH monoclonal antibody	Cell Signaling Technology
Mouse Anti-TGF-β1 Monoclonal Antibody	Abcam (Cambridge, UK)
Rabbit anti-p-SMAD2/3 polyclonal antibody	Abcam
Mouse secondary antibody, rabbit secondary antibody	Thermo Scientific (Waltham, MA, USA)

**Table 4:** Primer sequences for mice.

Primer Name	Sequence	Length
TIMP1-F	GGCATCTGGCATCCTCTTGTTG	22
TIMP1-R	AAGGTGGTCTCGTTG ATTTCTGG	23
Col3-F	GACAACTGATGGTGCTACTCTGAG	24
Col3-R	AGTGGGATGAAGAAGGGTGAGAAG	24
Col1-F	CTGACTGGAAGAGCGGAGAGTAC	23
Col1-R	AGTAGGGAACACACAGGTCTGAC	23
TGFβ1-F	AGCAACAATTCCTGGCGTTACC	22
TGFβ1-R	GTATTCCGTCTCCTTGGTTCAGC	23
MMP1-F	CCAAATCCCATCCAGCCAACAG	22
MMP1-R	CCCGAATGTAGAACCTGCCTTTG	23
GAPDH-F	GCAAATTCAACGGCACAGTCAAG	23
GAPDH-R	ACACCAGTAGACTCCACGACATAC	24

## Data Availability

Data will be made available on request.
